# FLI-1 is expressed in a wide variety of hematolymphoid neoplasms: a special concern in the differential diagnosis

**DOI:** 10.1007/s10238-023-01284-x

**Published:** 2024-01-27

**Authors:** Uiju Cho, Hee Jeong Cha, Hyun Jung Kim, Soo Kee Min, Hee Kyung Kim, Hye Ra Jung, Gyeongsin Park, Ji Eun Kim

**Affiliations:** 1 Department of Pathology, St. Vincent’s Hospital, College of Medicine, The Catholic University of Korea, Seoul, Republic of Korea; 2 Department of Pathology, Ulsan University Hospital, University of Ulsan College of Medicine, Ulsan, Republic of Korea; 3 Department of Pathology, Sanggye Paik Hospital, Inje University, Seoul, Republic of Korea; 4 Department of Pathology, Chung-ang University Gwangmyeong Hospital, Gwangmyeong, Republic of Korea; 5 Department of Pathology, Soonchunhyang University Hospital, Bucheon, Republic of Korea; 6 Department of Pathology, Keimyung University School of Medicine, Daegu, Republic of Korea; 7 Department of Pathology, Seoul St. Mary’s Hospital, College of Medicine, The Catholic University of Korea, 222 Banpodaero, Seocho-gu, Seoul, 06591 Republic of Korea; 8 Department of Pathology, Seoul National University Boramae Hospital, 20 Boramae-Ro 5-Gil, Dongjak-Gu, Seoul, 07061 Republic of Korea

**Keywords:** FLI-1, Plasmablastic lymphoma, Hematologic neoplasm, Lymphoma, Biomarker

## Abstract

Friend Leukemia Virus Integration 1 (FLI-1) is a member of E26 transformation-specific family of transcription factors that participates in hematopoietic and vascular endothelial cell development. Immunohistochemical detection of FLI-1 has been widely used to diagnose vascular tumors or, more evidently, Ewing’s sarcoma. However, the expression pattern of FLI-1 in hematolymphoid neoplasms remains unclear. Therefore, in this study, we aimed to investigate the expression of FLI-1 in these tumors, focusing on high-grade lesions, which presents a diagnostic challenge by mimicking Ewing’s sarcoma. We evaluated the expression FLI-1 in various types of lymphoid and plasmacytic tumors, including 27 plasmablastic lymphomas, 229 diffuse large B-cell lymphomas, 22 precursor T- or B-lymphoblastic lymphomas, 24 angioimmunoblastic-type nodal T-follicular helper cell lymphomas, 52 peripheral T-cell lymphomas, NOS, 18 Burkitt lymphomas, 18 non-gastric lymphomas of mucosa-associated lymphoid tissue, 38 chronic lymphocytic leukemia/small lymphocytic lymphomas, 15 mantle cell lymphomas, 23 gastric MALT lymphomas, 50 plasma cell myelomas, and 38 follicular lymphomas. We calculated the H-scores of FLI-1 immunostaining, ranging from 0 to 200, and used the scores to analyze the clinicopathological significance of FLI-1 statistically. FLI-1 was expressed to varying degrees in all types of hematological tumors. FLI-1 expression was detected in 84.1% of patients (466/554). FLI-1 was highly expressed in precursor T- or B-lymphoblastic lymphomas. Follicular lymphomas exhibited low FLI-1 expression. In plasmablastic lymphoma, 85.2% of the patients were focally positive for FLI-1. FLI-1 expression did not correlate with clinicopathological variables, such as demographic data or disease stage, in patients with plasmablastic lymphoma and diffuse large B-cell lymphoma. However, FLI-1 overexpression was associated with poorer overall survival in patients with plasmablastic lymphoma. This study demonstrates that FLI-1 is expressed in various hematolymphoid neoplasms. FLI-1 expression can lead to diagnostic confusion, especially in small blue round cell tumors, such as lymphoblastic lymphoma, plasmablastic lymphoma, and plasma cell myeloma, when distinguishing tumors positive for CD99 and CD56 without CD3, CD20, or CD45. Our findings also suggested the possibility of FLI-1 as a potential prognostic biomarker for plasmablastic lymphoma.

**Supplementary Information:**

The online version contains supplementary material available at 10.1007/s10238-023-01284-x.

## Introduction

FLI-1, a member of the E26 transformation-specific (ETS) family of transcription factors, is expressed primarily in hematopoietic cells, including most cells active in immunity, and is involved in immune cell development, maintaining immune homeostasis [1]. FLI-1 is a transcription factor named for Friend leukemia virus integration site 1, of which the animal heterology of this gene is targeted for retroviral integration [2, 3]. One year after its discovery, FLI-1 was found to be a target of translocation in most of Ewing’s sarcomas; the most common translocation is *t*(11, 22). This translocation forms a fusion protein, EWS-FLI-1, that exhibits strong tumorigenic activity [4].

**Fig. 1:**
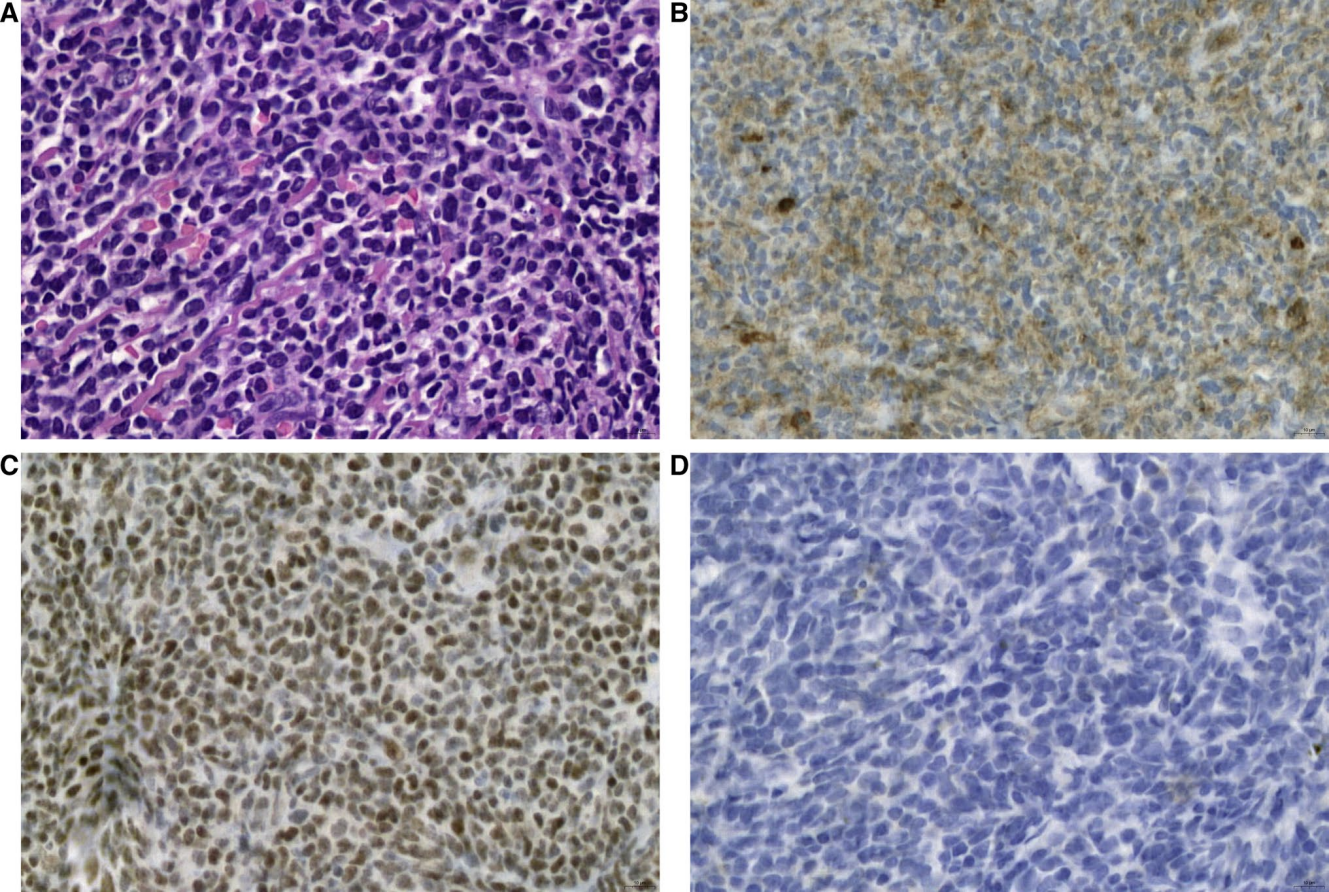
Micrographs of a non-secretory type solitary intraosseous plasmacytoma mimicking Ewing’s sarcoma. This small blue round cell lesion was in a bone without discernable histologic differentiation (**A**, hematoxylin and eosin stain). The tumor cells were weakly positive for CD99 (**B**). The tumor cells also expressed FLI-1 (**C**) and were negative for leukocyte common antigen (LCA) (**D**) (all × 1000)

In addition, expression of FLI-1 has been demonstrated in vascular neoplasms such as angiosarcoma and hemangioendothelioma [5]. Consequently, FLI-1 is a diagnostic marker for Ewing’s sarcoma in small blue round cell and vascular origin tumors [5, 6]. A limited number of studies have investigated the expression of FLI-1 in hematolymphoid neoplasms. Folpe et al. reported FLI-1 positivity in 7/8 (88%) cases of lymphoblastic lymphoma [5]. Subsequently, a few additional studies investigated FLI-1 expression in hematolymphoid neoplasms [7–10]; however, most of the studies included small sample sizes. The largest study included 132 non-Hodgkin’s lymphoma cases, and 14.4% of the lymphomas demonstrated FLI-1 expression, respectively [10]. Recently, we identified a non-secretory solitary plasmacytoma of the thoracic spine that closely mimicked Ewing’s sarcoma in both morphological and immunohistochemical phenotypes. It exhibited strong FLI-1 and CD99 expression but lacked leukocyte common antigen (LCA) expression, leading to diagnostic confusion (Fig. 1). This index case confirms that the expression of FLI-1 in hematolymphoid neoplasms has not been sufficiently explored, specifically in plasmacytic or high-grade lesions that mimic Ewing’s sarcoma.

Therefore, in this study, we aimed to investigate the expression of FLI-1 in hematolymphoid neoplasms, focusing on high-grade lesions that mimic small round cell tumors. To the best of our knowledge, our study has researched the largest sample size to date. We also aimed to determine the clinicopathological significance and diagnostic value of FLI-1.

## Materials and methods

### Patients and tissue samples

Between 2007 and 2017, hematolymphoid neoplasm tissue samples were obtained from 554 patients diagnosed with hematolymphoid neoplasms at one of eight university hospitals in Korea. Two experienced hematopathologists (UC and JEK) confirmed the diagnosis, according to the 5th edition of the World Health Organization Classification of Hematolymphoid Tumors [11]. Clinical data, including overall survival (OS), were collected from the electronic medical records. For diffuse large B-cell lymphoma (DLBCL), the Hans algorithm was used to determine the cell-of-origin (COO) based on CD10, BCL6, and MUM1 immunohistochemistry (IHC) at the time of diagnosis. This study was approved by the Institutional Review Board of the Catholic University Catholic Medical Center (IRB No. XC22SIDI0076) and Seoul National University Boramae Hospital (IRB No. 10-2022-54).

### Evaluation of immunohistochemical staining

In DLBCL, tissue microarray blocks were constructed with a pair of 2–5 mm-wide tumor cores from the original paraffin blocks and used for IHC. In other cases, the whole tissue section was used. IHC for FLI-1 (clone G146-222, mouse monoclonal, 1:200; BD Biosciences, San Jose, CA, USA) was performed using a BenchMark ULTRA automated staining platform (Ventana Medical Systems, Tucson, AZ, USA), according to the manufacturer’s recommendations. Heat-induced epitope retrieval was performed using the Ventana CC1 mild reagent and OptiView detection kit (Ventana Medical Systems), according to the manufacturer’s instructions. FLI-1 expression was semi-quantitatively estimated using the H-score with a three-tiered score (0, absent; 1, weak-to-moderate; 2, strong nuclear staining) multiplied by the frequency (%) of positive tumor cells, ranging from 0 to 100. The intensity score was defined as follows: score 0, no staining; score 1, weaker nuclear staining than the internal endothelial cell control staining; and score 2, same as or stronger nuclear staining than the internal endothelial cell control staining (Supplementary Fig. 1). Cases without a positive internal control, that is, the endothelium, were not scored and were excluded from the study. The dichotomized analysis defined cases with at least 10% weak nuclear staining intensity as “positive.”

### Statistical analysis

Categorical variables are presented as numbers (percentages); whereas, continuous variables are expressed as means (standard deviations) or medians (ranges). Pearson’s chi-square or Fisher’s exact test was used to compare categorical variables across groups. Mean values were compared across multiple groups using ANOVA, and post hoc analyses were adjusted using the Bonferroni method. Overall survival was defined as the time elapsed between the date of diagnosis and death. Kaplan–Meier survival curves were constructed to compare survival rates between the two groups, and the log-rank ratio was derived from the survival data. All statistical analyses were performed using R version 4.2.2. Statistical significance was set at a two-tailed *p *value < 0.05.

## Results

### Nuclear expression of FLI-1 in hematolymphoid neoplasms

**Fig. 2:**
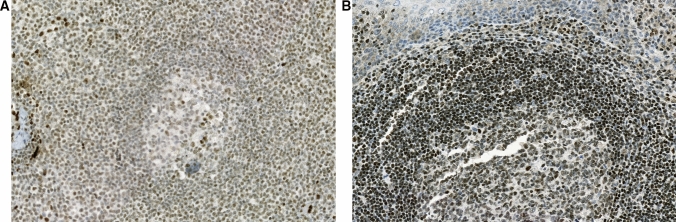
FLI-1 immunohistochemical staining in a palatine tonsil (**A**) and a reactive lymph node (**B**). All lymphocytes in T- and B-cell zones express FLI-1 to varying degrees. Endothelial cells lining the vessels exhibit strong staining intensity (all × 400)

In reactive lymph nodes and palatine tonsils, lymphocytes in both the T- and B-cell zones showed nuclear FLI-1 with varying intensities. Expression was weaker in the germinal center and slightly stronger in the mantle and interfollicular zones. Compared to the endothelial cells of the vessels, the reactive lymph nodes and palatine tonsil lymphocytes exhibited similar or weaker staining intensities (Fig. 2a and b). Nuclear FLI-1 expression was detected in 84.1% (466/554) of the patients (positive H-score > 10). FLI-1 expression was assessed in a wide range of hematologic tumors, including 24 angioimmunoblastic-type nodal T-follicular helper cell lymphomas (angioimmunoblastic T-cell lymphoma), 18 Burkitt lymphomas, 38 chronic lymphocytic leukemia/small lymphocytic lymphomas, 229 DLBCL (not otherwise specified), 38 follicular lymphomas, 23 gastric extranodal marginal zone lymphomas of mucosa-associated lymphoid tissue (MALT lymphomas), 18 non-gastric MALT lymphomas, 22 precursor T- or B-lymphoblastic leukemia/lymphomas, 15 mantle cell lymphomas, 28 peripheral T-cell lymphomas, NOS, 50 plasma cell myelomas, and 27 plasmablastic lymphomas (PBL).

**Fig. 3:**
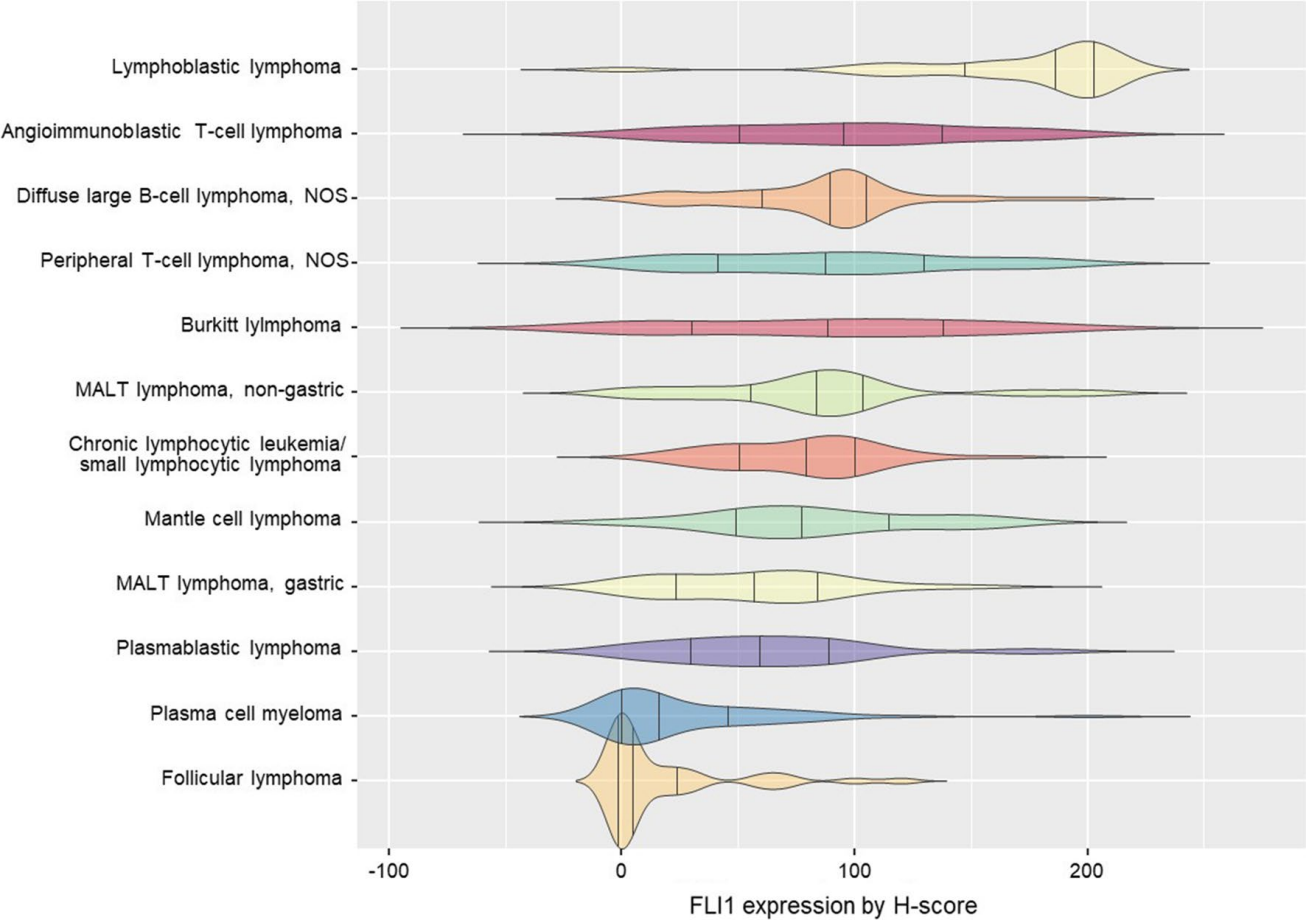
Expression pattern in various hematolymphoid neoplasms

**Fig. 4:**
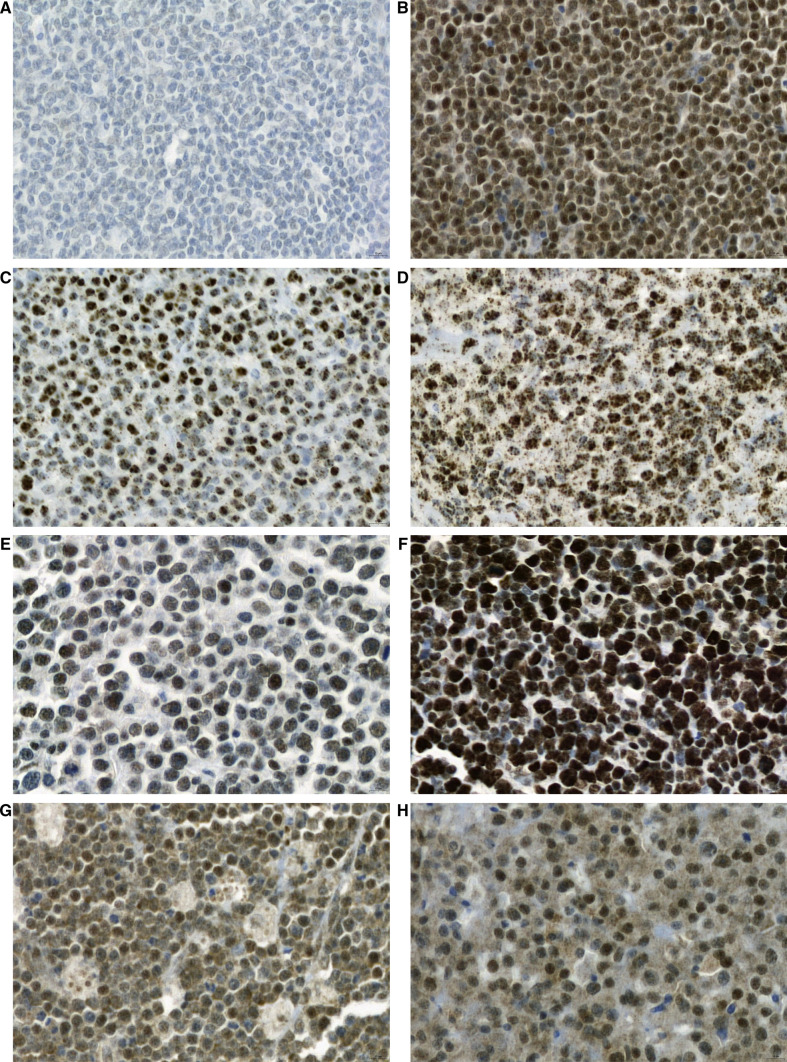
FLI-1 expression in hematolymphoid neoplasms. Lymphoblastic lymphoma (**A**), follicular lymphoma (**B**), plasmablastic lymphoma (**C** and **D**), diffuse large B-cell lymphoma, NOS (**E** and **F**), Burkitt lymphoma (**G**), and plasma cell myeloma (**H**). (all × 1000)

Nuclear FLI-1 expression in various hematolymphoid neoplasms is shown in Fig. 3. FLI-1 expression tended to be heterogeneous, even within the same neoplasm. However, in lymphoblastic and follicular lymphomas, uniformly strong and diffuse or extremely low contrast FLI-1 expression was observed (Fig. 4a and b). Precursor T- or B- lymphoblastic lymphoma exhibited the highest FLI-1 expression (mean FLI-1 H-score: 168.6, standard deviation: 50.5) and was positive in 95.5% of cases (all but one case) (Table 1). In contrast, follicular lymphoma had the lowest FLI-1 H-score (mean: 17.2, standard deviation: 30.5), with a 34.2% positivity rate. The cohort had 25 grade 1–2, 12 grade 3A, and one grade 3B follicular lymphoma. Among the FLI-1 positive FL group, nine were grade 1–2, and four were grade 3. FLI-1 negative group comprised 16 grade 1–2 FL and nine grade 3 FL. The association between FLI-1 expression status and FL grade (grade 1–2 vs grade 3) was insignificant (*p* = 0.7471).

**Table 1:** FLI-1 expression in various hematolymphoid neoplasms

Diagnosis	*n*	FLI-1 expression (H-score)		FLI-1 expression positivity^‡^ (total *n* = 468)
		Mean (standard deviation)^*^	Median (range)	
Precursor T- or B- lymphoblastic lymphoma	22	168.64 (± 50.45)	200 (0–200)	21 (95.5%)
Angioimmunoblastic T-cell lymphoma^†^	24	95.21 (± 53.98)	95 (5–184)	23 (95.8%)
Diffuse large B-cell lymphoma, NOS	229	86.92 (± 42.60)	91.27 (0–200)	218 (95.2%)
Peripheral T-cell lymphoma, NOS	52	88.41 (± 54.74)	90 (5–185)	49 (94.2%)
Burkitt lymphoma	18	85.56 (± 62.66)	87.5 (0–180)	13 (72.2%)
MALT lymphoma, non-gastric	18	83.33 (± 48.87)	85 (0–200)	16 (88.9%)
Chronic lymphocytic leukemia/small lymphocytic lymphoma	38	76.91 (± 32.92)	82.5 (15–165)	38 (100.0%)
Mantle cell lymphoma	15	82.00 (± 44.75)	80 (0–155)	14 (93.3%)
MALT lymphoma, gastric	23	56.30 (± 38.87)	62.5 (0–150)	19 (82.6%)
Plasmablastic lymphoma	27	63.15 (± 44.55)	60 (0–180)	23 (85.2%)
Plasma cell myeloma	50	27.30 (± 39.06)	10 (0–200)	21 (42.0%)
Follicular lymphoma	38	17.24 (± 30.46)	0 (0–120)	13 (34.2%)

NOS, not otherwise specified

^†^Angioimmunoblastic-type nodal T-follicular helper cell lymphomas in 5th edition WHO classification of haematolymphoid neoplasms

^*^P value < 0.0001 for heterogeneity. The post hoc analysis results are presented in Supplementary Table 1

^‡^Cutoff value for positivity is 10%

For PBL, at least focally, 85.2% of cases expressed FLI-1, but the intensity was generally weak to moderate (Fig. 4c). Only two cases exhibited strong and diffuse FLI-1 expression, as observed in lymphoblastic lymphoma (Fig. 4d). The mean DLBCL H-score was 86.92 (standard deviation: 42.6). Most DLBCLs were positive for FLI-1 with varying degrees of intensity (Fig. 4e and f), and only a few cases exhibited strong and diffuse expression.

Lymphoma types with overall intermediate intensity or showing very heterogenous expression pattern were angioimmunoblastic T-cell lymphoma (H-score 95.21 ± 53.98), peripheral T-cell lymphoma, NOS (H-score 88.41 ± 54.74), Burkitt lymphoma (H-score 85.56 ± 62.66), gastric and non-gastric MALT lymphomas (H-score 56.30 ± 38.87 and 83.33 ± 48.87, respectively), chronic lymphocytic leukemia/small lymphocytic lymphoma (H-score 76.91 ± 32.92) and mantle cell lymphoma (H-score 82.00 ± 44.75). Plasma cell myeloma demonstrated low range FLI-expression (H-score 27.30 ± 39.06).

### Clinicopathologic significance of nuclear FLI-1 expression in plasmablastic lymphoma and diffuse large B-cell lymphoma

**Fig. 5:**
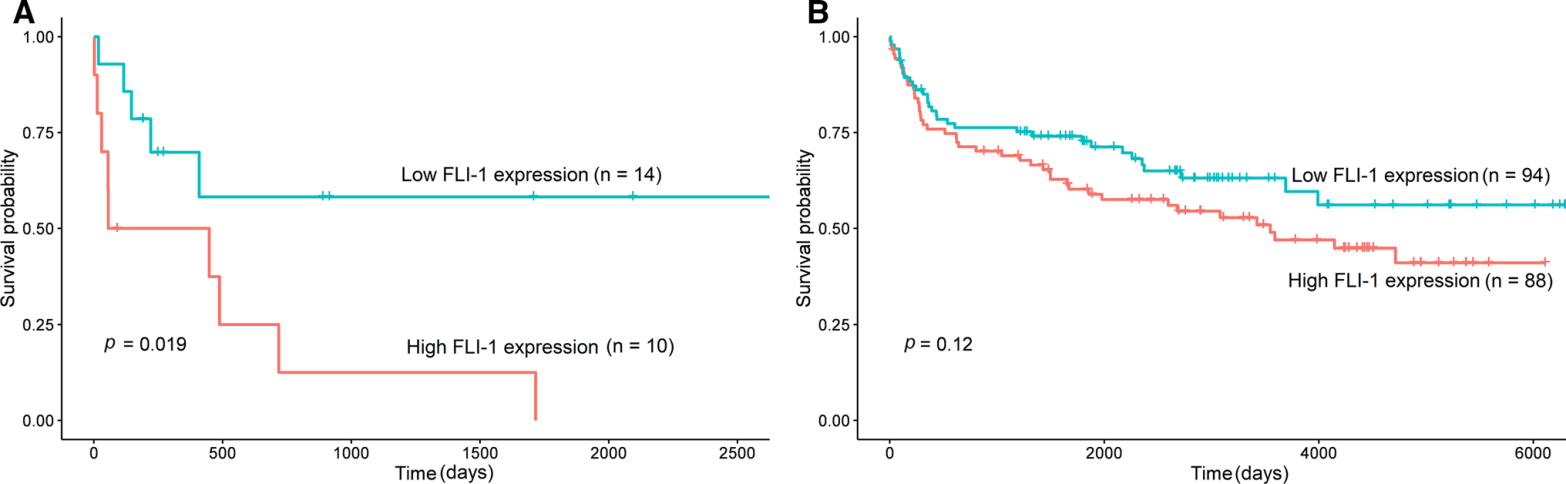
Kaplan–Meier survival curve of high-grade B-cell lymphomas. High FLI-1 expression showed poorer overall survival in patients with plasmablastic lymphoma (*p* = 0.019) (**A**). FLI-1 expression was not associated with overall survival in diffuse large B-cell lymphoma, NOS (*p* = 0.120) (**B**)

**Fig. 6:**
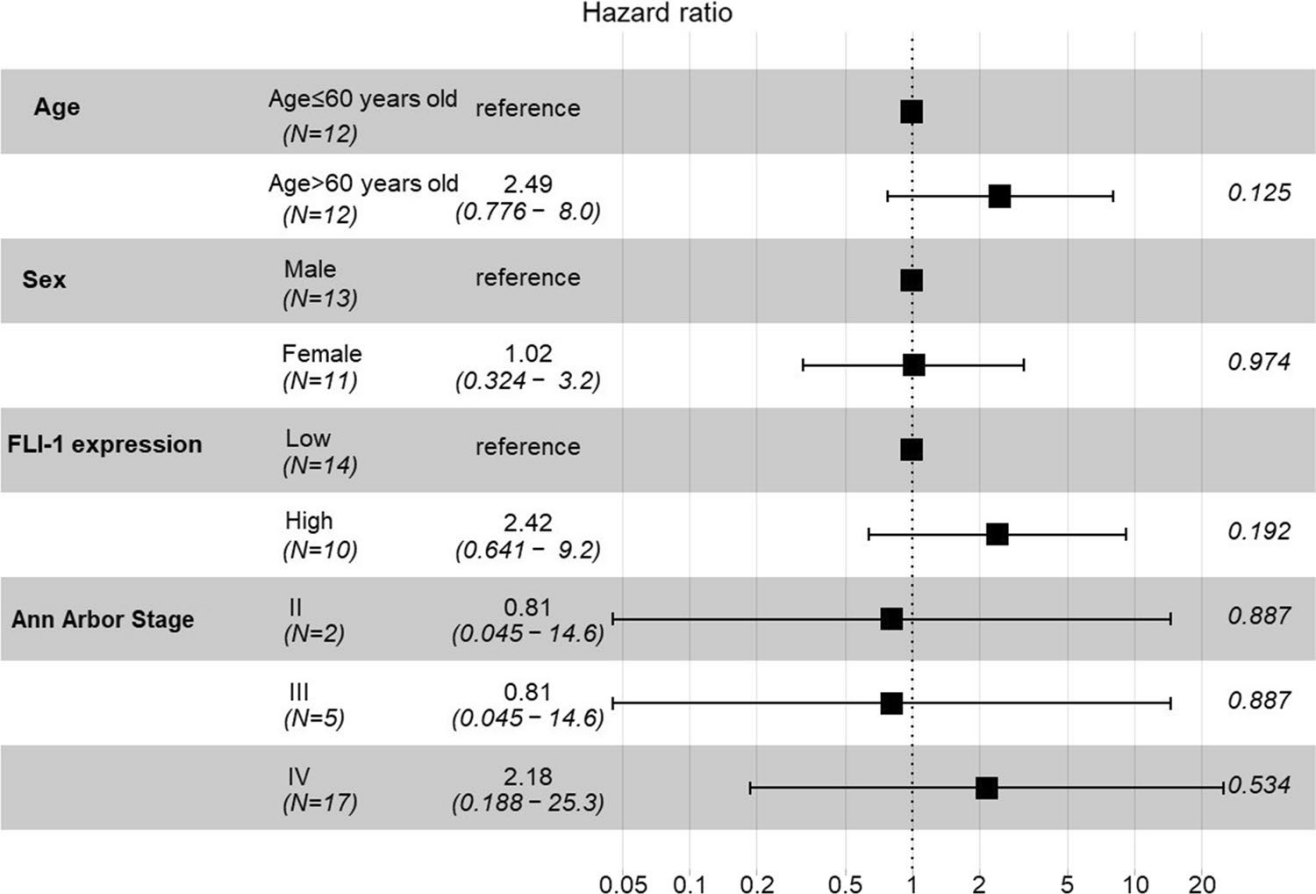
Multivariate overall survival analysis in patients with plasmablastic lymphoma

The association between various clinicopathologic parameters and FLI-1 expression was assessed in PBL (*N* = 24) and DLBCL (*N* = 182). Patients’ demographic data and correlation results are summarized in Table 2. The median follow-up periods for patients with PBL and DLBCL were 234.5 days (2–2660 days) and 1902 days (3–6809 days). During the follow-up period, 58.3% (14/24) and 42.3% (77/182) of patients with PBL and DLBCL succumbed to the disease, respectively. FLI-1 expression did not correlate with patient age, sex, COO, or Ann Arbor stage in either PBL or DLBCL (all *p* > 0.05). Univariate survival analysis, according to FLI-1 expression dichotomized by a median H-score cutoff (60 for PBL and 91 for DLBCL), revealed that high FLI-1 expression was associated with poorer overall survival in patients with PBL (log-rank test, *p* = 0.019) (Fig. 5a). The 2-year survival rate of patients with high FLI-1 expression was significantly lower (12.5%) than that of patients with low FLI-1 expression (58.2%). However, in multivariate survival analysis adjusted for sex, age, and stage, FLI-1 expression was not an independent prognostic factor (*p* = 0.192) (Fig. 6). In patients with DLBCL, FLI-1 expression was not associated with overall survival (log-rank *p* = 0.120) (Fig. 5b).

**Table 2:** Correlation between FLI-1 expression and clinicopathologic factors in patients with plasmablastic lymphoma and diffuse large B-cell lymphoma, NOS

Characteristics	Plasmablastic lymphoma			Diffuse large B-cell lymphoma		
	Low FLI-1 (*n* = 14)	High FLI-1 (*n* = 10)	*P *value	Low FLI-1 (*n* = 94)	High FLI-1 (n = 88)	P value
*Sex*						
Male	7 (50.0%)	6 (60.0%)	0.945	55 (58.5%)	48 (54.5%)	0.697
Female	7 (50.0%)	4 (40.0%)		39 (41.5%)	40 (45.5%)	
*Age*						
> 60 years old	6 (42.9%)	6 (60.0%)	0.679	42 (44.7%)	48 (54.5%)	0.237
≤ 60 years old	8 (57.1%)	4 (40.0%)		52 (55.3%)	40 (45.5%)	
*Ann Arbor stage*						
I	0 (0.0%)	0 (0.0%)	0.194	22 (23.4%)	21 (23.9%)	0.398
II	2 (14.3%)	0 (0.0%)		30 (31.9%)	19 (21.6%)	
III	4 (28.6%)	1 (10.0%)		17 (18.1%)	22 (25.0%)	
IV	8 (57.1%)	9 (90.0%)		25 (26.6%)	26 (29.5%)	
*COO phenotype*						
GCB	−	−		33 (35.1%)	29 (33.0%)	0.881
Non-GCB	−	−		61 (64.9%)	59 (67.0%)	

*NOS* not otherwise specified, *COO* cell of origin, *GCB* germinal center B-cell type

## Discussion

In this study, we evaluated FLI-1 expression in various hematolymphoid neoplasms to determine the diagnostic specificity and clinical significance of FLI-1 expression in PBL. FLI-1 was expressed in a wide variety of hematologic tumors, most frequently in lymphoblastic lymphoma, which was not elucidated previously.

Although FLI-1 was initially discovered as a causative factor of lymphoma-related diseases [3], only a limited number of studies have focused on the clinical significance of FLI-1 in hematolymphoid neoplasms. In contrast, FLI-1 expression has been well established in Ewing’s sarcoma and vascular tumors; therefore, it serves as a useful diagnostic marker for these tumor types [5, 6]. Thus, in cases where a small round cell tumor is present where Ewing’s sarcoma is prevalent, such as the bone marrow, the diagnosis is skewed toward Ewing’s sarcoma when based solely on FLI-1 expression. Hematolymphoid tumor diagnosis is largely overlooked.

FLI-1 plays a crucial role in developing and differentiating lymphoid and myeloid lineage tissues by enhancing the promoter or enhancer activity of genes encoding growth factor receptors and integrin families. However, the mechanisms underlying hematolymphoid neoplasm’s down- or up-regulation remain unclear [12]. In our study, at least at low levels, FLI-1 was expressed in most hematolymphoid neoplasms (84.1%). Only a small number of studies have examined FLI-1 expression in hematological lesions. According to Mhawech–Fauceglia et al., the rate of FLI-1 expression is extremely low in non-Hodgkin’s lymphomas, except for Burkitt lymphoma (2/2) [10]. Another study published contradictory results, demonstrating significantly high FLI-1 positivity in several T-cell lymphomas, including precursor lymphoblastic lymphoma/leukemia and other mature T-cell lymphomas [9, 10]. Notably, these studies were conducted before 2007; therefore, the quality of the antibody and the antibody clone used currently differs from that used in the previous studies. Thus, the observed discrepancies could be attributed to antibody differences. To date, our study included the largest sample size and has been conducted on almost all hematolymphoid and plasmacytic tumor types.

Interestingly, in the current study, we observed that FLI-1 expression was higher in high-grade or precursor-origin lymphomas than in low-grade lymphomas, such as MALT lymphoma and follicular lymphoma (Fig. 3). Previous studies have consistently demonstrated FLI-1 overexpression in lymphoblastic lymphoma cases, albeit in small sample groups (88%, 7/8; 92%, 33/36) [5, 9]. Acute lymphoblastic lymphoma/leukemia resembles Ewing sarcoma in terms of morphology and clinical characteristics, especially in cases that are CD45-negative and CD99-positive [13]. Consequently, FLI-1 expression in acute lymphoblastic lymphoma/leukemia has been evaluated more extensively than in other hematolymphoid neoplasms. However, the cause of FLI-1 overexpression in lymphoblastic lymphoma has not yet been elucidated [5, 9]. In erythroleukemic cells, FLI-1 overexpression dedifferentiates cells to an earlier progenitor state; whereas, Spi-1/PU.1 (another ETS factor) promotes erythroid cell maturation. This highlights the importance of FLI-1 in maintaining the balance between erythroid differentiation and proliferation [14]. In acute myeloid leukemia, FLI-1 suppression inhibits cell growth and induces cell death, indicating that FLI-1 plays a crucial role in the survival and maintenance of leukemic cells. FLI-1 overexpression is an adverse prognostic factor for AML [15, 16]. In a mouse model, FLI-1 overexpression resulted in the development of pre-T-cell lymphoblastic leukemia/lymphoma, associated with increased NOT1 expression [17]. FLI-1 is a novel upstream transcriptional activator of NANOGP2 in T-lymphocyte leukemia cells, indicating its involvement in leukemogenesis [18].

The biology of FLI-1 in cancer remains poorly understood, and only a limited number of studies have investigated its role [19–21]. Deregulated expression or formation of chimeric fusion proteins of the ETS family, owing to proviral insertions or chromosomal translocations, is associated with leukemia and specific solid tumor types [22]. The ETS family of transcription factors has been implicated in malignant transformation and tumor progression through oncogene and tumor suppressor gene regulation. Moreover, they have been linked to tumor angiogenesis, invasion, and metastasis, often correlating with poor survival outcomes [22–25]. In ovarian carcinoma, FLI-1 is expressed in 74% of cases and is specifically confined to malignant cells [19]. High FLI-1 expression is significantly associated with advanced tumor stage, lymph node metastasis, and poor patient survival [19]. FLI-1 is a predictor of poor prognosis in breast and endometrial cancers and acute myeloid leukemia [15, 16, 26, 27]. Therefore, FLI-1 has been identified as a potential anticancer therapeutic target for various tumors [21]. In our study, the prognosis in the PBL FLI-1 overexpression group was significantly worse than that in the low FLI-1 expression group (2-year survival rate 12.5 vs. 58.2, *p* = 0.019), indicating the potential utilization of FLI-1 as a prognostic marker. As the biological role of FLI-1 in PBL remains unclear, further investigations are needed to determine the clinical value of FLI-1 and the mechanisms underlying its association with survival.

Occasionally, the pathological differentiation between plasma cell myeloma (PCM) and PBL is unreliable. In our study, FLI-1 expression in PCM and PBL was similar to that observed in other groups. In addition, there was no significant difference in the FLI-1 expression score between the two groups (PCM and PBL). However, PBL exhibited a higher average FLI-1 expression than PCM (mean FLI-1 H-score 27.30 ± 39.06 vs. 63.15 ± 44.55, respectively; *p* = 0.0347). Despite this finding, the FLI-1 staining pattern varied greatly within each disease entity, as shown in Fig. 2, indicating that FLI-1 staining is not helpful in distinguishing between PCM and PBL, nor is it useful for differentiating PBL from DLBCL. Considering the FLI-1 expression pattern alone, these entities had considerable overlap, indicating that FLI-1 staining is not an appropriate diagnostic tool in these cases (Fig. 2).

The expression of FLI-1 can lead to serious diagnostic confusion, especially in small blue round cell tumors, when distinguishing tumors positive for CD99 and CD56 without CD3, CD20, or CD45, such as lymphoblastic lymphoma, PBL, and PCM. In particular, non-secretory intraosseous PCM closely mimics Ewing’s family tumors, as in our index case. Diagnosing small blue round cell tumors requires a large panel of immunohistochemical markers and molecular studies. Therefore, expression of FLI-1 alone will not lead to diagnosing sarcoma in normal situations. However, a lack of knowledge on FLI-1 expression in lymphoma could cause a great deal of detour in the journey to the correct diagnosis.

The major limitation of this study is it lacks molecular characterization of the FLI-1 positive lymphomas. Molecular studies, such as fluorescence in situ hybridization or next-generation sequencing for FLI-1 translocation, amplification, or analysis of FLI-1 expression level through mRNA level, are needed in the future to elucidate the biology of FLI-1 in lymphomas.

## Conclusion

This study augments our understanding of FLI-1 expression in hematolymphoid neoplasms by analyzing several cases spanning various diagnoses. This is the first study to illustrate FLI-1 expression in PBL. Recognition of FLI-1 positivity in high-grade hematolymphoid neoplasms, including PBL, could prompt a more careful approach to diagnosing small round cell tumors and can help avoid erroneous diagnoses when differentiating these neoplasms. Moreover, our findings suggest the possibility of FLI-1 as a potential prognostic biomarker for PBL. Further research with a larger cohort will help discover the prognostic value of FLI-1 in lymphomas.

## Supplementary Information

Below is the link to the electronic supplementary material.

Supplementary file1 (PDF 268 kb)

## Declarations

### Availability of data and materials

The dataset supporting the conclusions of this article is included within the report.

## References

[1] Gallant S, Gilkeson G. ETS transcription factors and regulation of immunity. Arch Immunol Ther Exp (Warsz). 2006;54:149–63. 10.1007/s00005-006-0017-z. 16652219 10.1007/s00005-006-0017-z

[2] Ben-David Y, Bani MR, Chabot B, De Koven A, Bernstein A. Retroviral insertions downstream of the heterogeneous nuclear ribonucleoprotein A1 gene in erythroleukemia cells: evidence that A1 is not essential for cell growth. Mol Cell Biol. 1992;12:4449–55. 10.1128/mcb.12.10.4449-4455.1992. 1406633 10.1128/mcb.12.10.4449-4455.1992 PMC360369

[3] Ben-David Y, Giddens EB, Letwin K, Bernstein A. Erythroleukemia induction by Friend murine leukemia virus: insertional activation of a new member of the ets gene family, Fli-1, closely linked to c-ets-1. Genes Dev. 1991;5:908–18. 10.1101/gad.5.6.908. 2044959 10.1101/gad.5.6.908

[4] Delattre O, Zucman J, Plougastel B, Desmaze C, Melot T, Peter M, et al. Gene fusion with an ETS DNA-binding domain caused by chromosome translocation in human tumors. Nature. 1992;359:162–5. 10.1038/359162a0. 1522903 10.1038/359162a0

[5] Folpe AL, Hill CE, Parham DM, O’Shea PA, Weiss SW. Immunohistochemical detection of FLI-1 protein expression: a study of 132 round cell tumors with emphasis on CD99-positive mimics of Ewing’s sarcoma/primitive neuroectodermal tumor. Am J Surg Pathol. 2000;24:1657–62. 10.1097/00000478-200012000-00010. 11117787 10.1097/00000478-200012000-00010

[6] Folpe AL, Chand EM, Goldblum JR, Weiss SW. Expression of Fli-1, a nuclear transcription factor, distinguishes vascular neoplasms from potential mimics. Am J Surg Pathol. 2001;25:1061–6. 10.1097/00000478-200108000-00011. 11474291 10.1097/00000478-200108000-00011

[7] Nilsson G, Wang M, Wejde J, Kreicbergs A, Larsson O. Detection of EWS/FLI-1 by immunostaining. An adjunctive tool in diagnosis of Ewing’s sarcoma and primitive neuroectodermal tumour on cytological samples and paraffin-embedded archival material. Sarcoma. 1999;3:25–32. 10.1080/13577149977839. 18521261 10.1080/13577149977839 PMC2395406

[8] Llombart-Bosch A, Navarro S. Immunohistochemical detection of EWS and FLI-1 proteinss in Ewing sarcoma and primitive neuroectodermal tumors: comparative analysis with CD99 (MIC-2) expression. Appl Immunohistochem Mol Morphol. 2001;9:255–60. 10.1097/00129039-200109000-00010. 11556754 10.1097/00129039-200109000-00010

[9] Lin O, Filippa DA, Teruya-Feldstein J. Immunohistochemical evaluation of FLI-1 in acute lymphoblastic lymphoma (ALL): a potential diagnostic pitfall. Appl Immunohistochem Mol Morphol. 2009;17:409–12. 10.1097/PAI.0b013e3181972b6d. 19349856 10.1097/PAI.0b013e3181972b6d

[10] Mhawech-Fauceglia P, Herrmann FR, Bshara W, Odunsi K, Terracciano L, Sauter G, et al. Friend leukaemia integration-1 expression in malignant and benign tumours: a multiple tumour tissue microarray analysis using polyclonal antibody. J Clin Pathol. 2007;60:694–700. 10.1136/jcp.2006.039230. 16917000 10.1136/jcp.2006.039230 PMC1955051

[11] Alaggio R, Amador C, Anagnostopoulos I, Attygalle AD, Araujo IBO, Berti E, et al. The 5th edition of the World Health Organization classification of hematolymphoid tumors: lymphoid neoplasms. Leukemia. 2022;36:1720–48. 10.1038/s41375-022-01620-2. 35732829 10.1038/s41375-022-01620-2 PMC9214472

[12] Li Y, Luo H, Liu T, Zacksenhaus E, Ben-David Y. The ets transcription factor Fli-1 in development, cancer, and disease. Oncogene. 2015;34:2022–31. 10.1038/onc.2014.162. 24909161 10.1038/onc.2014.162 PMC5028196

[13] Lucas DR, Bentley G, Dan ME, Tabaczka P, Poulik JM, Mott MP. Ewing sarcoma vs lymphoblastic lymphoma. A comparative immunohistochemical study. Am J Clin Pathol. 2001;115:11–7. 10.1309/K1XJ-6CXR-BQQU-V255. 11190795 10.1309/K1XJ-6CXR-BQQU-V255

[14] Vecchiarelli-Federico LM, Liu T, Yao Y, Gao Y, Li Y, Li YJ, et al. Fli-1 overexpression in erythroleukemic cells promotes erythroid de-differentiation while Spi-1/PU.1 exerts the opposite effect. Int J Oncol. 2017;51:456–66. 10.3892/ijo.2017.4027. 28586009 10.3892/ijo.2017.4027 PMC5505126

[15] Yan X, Yu Y, Li L, Chen N, Song W, He H, et al. Friend leukemia virus integration 1 is a predictor of poor prognosis of breast cancer and promotes metastasis and cancer stem cell properties of breast cancer cells. Cancer Med. 2018;7:3548–60. 10.1002/cam4.1589. 29869379 10.1002/cam4.1589 PMC6089157

[16] Kornblau SM, Qiu YH, Zhang N, Singh N, Faderl S, Ferrajoli A, et al. Abnormal expression of FLI1 protein is an adverse prognostic factor in acute myeloid leukemia. Blood. 2011;118:5604–12. 10.1182/blood-2011-04-348052. 21917756 10.1182/blood-2011-04-348052 PMC3217360

[17] Smeets MFMA, Chan AC, Dagger S, Bradley CK, Wei A, Izon DJ. Fli-1 overexpression in hematopoietic progenitors deregulates T cell development and induces pre-T cell lymphoblastic leukaemia/lymphoma. PLoS ONE. 2013;8: e62346. 10.1371/journal.pone.0062346. 23667468 10.1371/journal.pone.0062346 PMC3646842

[18] Park SW, Do HJ, Choi W, Kim JH. Fli-1 promotes proliferation and upregulates NANOGP8 expression in T-lymphocyte leukemia cells. Biochimie. 2020;168:1–9. 10.1016/j.biochi.2019.10.005. 31626853 10.1016/j.biochi.2019.10.005

[19] Song W, Hu L, Li W, Wang G, Li Y, Yan L, et al. Oncogenic Fli-1 is a potential prognostic marker for the progression of epithelial ovarian cancer. BMC Cancer. 2014;14:424. 10.1186/1471-2407-14-424. 24923303 10.1186/1471-2407-14-424 PMC4089852

[20] Miao B, Bauer AS, Hufnagel K, Wu Y, Trajkovic-Arsic M, Pirona AC, et al. The transcription factor FLI1 promotes cancer progression by affecting cell cycle regulation. Int J Cancer. 2020;147:189–201. 10.1002/ijc.32831. 31846072 10.1002/ijc.32831

[21] Li L, Yu J, Cheng S, Peng Z, Luo H. Transcription factor Fli-1 as a new target for antitumor drug development. Int J Biol Macromol. 2022;209:1155–68. 10.1016/j.ijbiomac.2022.04.076. 35447268 10.1016/j.ijbiomac.2022.04.076

[22] Oikawa T, Yamada T. Molecular biology of the ets family of transcription factors. Gene. 2003;303:11–34. 10.1016/s0378-1119(02)01156-3. 12559563 10.1016/s0378-1119(02)01156-3

[23] Maroulakou IG, Bowe DB. Expression and function of ets transcription factors in mammalian development: a regulatory network. Oncogene. 2000;19:6432–42. 10.1038/sj.onc.1204039. 11175359 10.1038/sj.onc.1204039

[24] Davidson B, Reich R, Goldberg I, Gotlieb WH, Kopolovic J, Berner A, et al. Ets-1 messenger RNA expression is a novel marker of poor survival in ovarian carcinoma. Clin Cancer Res. 2001;7:551–7. 11297247

[25] Foos G, Hauser CA. Altered ets transcription factor activity in prostate tumor cells inhibits anchorage-independent growth, survival, and invasiveness. Oncogene. 2000;19:5507–16. 10.1038/sj.onc.1203946. 11114728 10.1038/sj.onc.1203946

[26] Scheiber MN, Watson PM, Rumboldt T, Stanley C, Wilson RC, Findlay VJ, et al. FLI1 expression is correlated with breast cancer cellular growth, migration, and invasion and altered gene expression. Neoplasia. 2014;16:801–13. 10.1016/j.neo.2014.08.007. 25379017 10.1016/j.neo.2014.08.007 PMC4212256

[27] Song W, Zhang T, Li W, Mu R, Zhang L, Li Y, et al. Overexpression of Fli-1 is associated with adverse prognosis of endometrial cancer. Cancer Invest. 2015;33:469–75. 10.3109/07357907.2015.1069831. 26305602 10.3109/07357907.2015.1069831

