# Supplementary figures and images for: FLI-1 is expressed in a wide variety of hematolymphoid neoplasms: a special concern in the differential diagnosis
Source: Clin Exp Med. 2024 Jan 27;24(1):18. doi: 10.1007/s10238-023-01284-x (PMC10821826; doi:10.1007/s10238-023-01284-x)

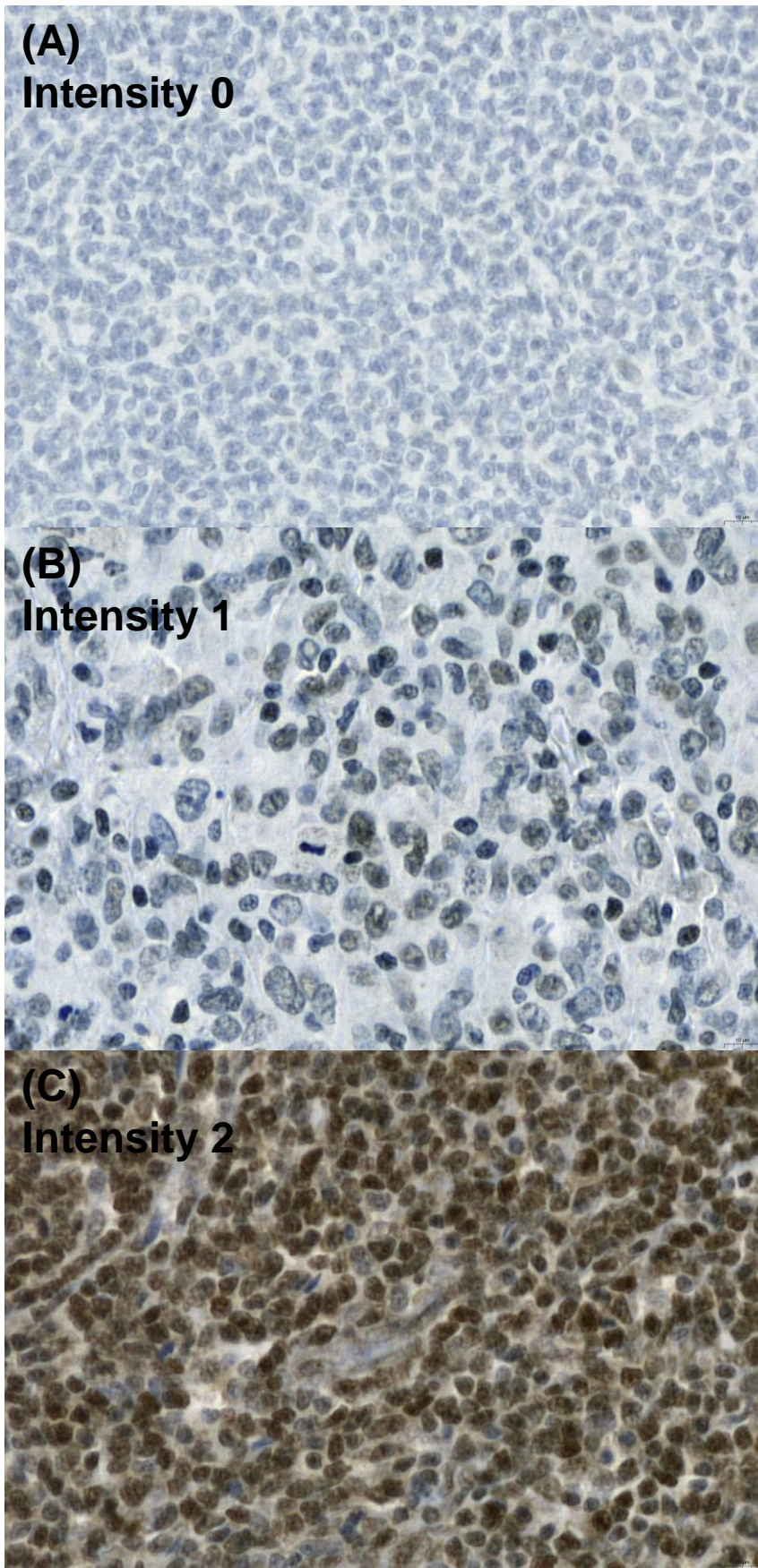

Supplementary Figure 1. Examples of the nuclear FLI-1 expression scored as intensity 0, 1, and 2.

Supplement: Supplementary file 1 — Supplementary file1 (PDF 268 kb) [file 10238_2023_1284_MOESM1_ESM.pdf]
